# Utilization of machine learning algorithm in the prediction of rehospitalization during one-year post traumatic spinal cord injury

**DOI:** 10.1038/s41393-024-01055-9

**Published:** 2025-02-15

**Authors:** Salma Aly, Yuying Chen, Abdulaziz Ahmed, Huacong Wen, Tapan Mehta

**Affiliations:** 1 Department of Family and Community Medicine, Heersink School of Medicine, University of Alabama at Birmingham, Birmingham, AL USA; 2 Department of Physical Medicine and Rehabilitation, Spain Rehabilitation Center, University of Alabama at Birmingham, Birmingham, AL USA; 3 Department of Health Services Administration, School of Health Professions, University of Alabama at Birmingham, Birmingham, AL USA

**Keywords:** Risk factors, Prognosis

## Abstract

**Study design:**

Retrospective cohort study.

**Objective:**

The primary aim was to develop a machine learning (ML) model to predict rehospitalization during the first year of traumatic spinal cord injury (TSCI) and to identify top predictors using data obtained during initial rehabilitation. The secondary aim was to predict prolonged hospital stay among the rehospitalized group.

**Setting:**

Eighteen SCI Model Systems centers throughout the United States.

**Methods:**

Data were retrieved from the National Spinal Cord Injury Model Systems Database. The participants were divided into 2 groups based on rehospitalization during the first year of injury. Those who experienced rehospitalization during first year were further grouped into prolonged stay (>75th quartile of the total length of stay) or non-prolonged stay. Variables considered in models included socio-demographic factors, clinical characteristics, and comorbidities.

**Results:**

The best performing classification models were Random Forest for predicting rehospitalization and Adaptive Boosting for prolonged stay. The most important predictors in both models were the degree of functional independence, American Spinal Injury Association (ASIA) scores, age, days from injury to rehabilitation admission and body mass index. Additionally, for prolonged stays, pressure injury as a reason for rehospitalization was top predictor.

**Conclusion:**

Functional Independence Measure (FIM) and ASIA scores emerge as key predictors of both rehospitalizations and prolonged rehospitalizations. These findings may assist clinicians in patient risk assessment. Furthermore, the identification of pressure injury as a primary predictor for prolonged stays signifies a targeted focus on preventive measures for pressure injury-related rehospitalizations, offering a specific strategy to enhance patient care and outcomes.

## Introduction

Traumatic spinal cord injury (TSCI) represents a significant health issue in the United States. According to the National Spinal Cord Injury Statistical Center (NSCISC), there are approximately 17,900 new cases annually [1]. Although TSCI is relatively uncommon compared to other chronic diseases, its consequences are impactful, and associated with physical and emotional difficulties for individuals, their families, and overall health and social systems.

One of the major problems confronting persons who sustain a TSCI is unplanned rehospitalization after initial rehabilitation care. The annual rates for these events range from 36% to 45% during the first year post injury [2]. According to NSCISC, the average length of rehospitalization stay during the first year of injury is 23 days [1]. The most common reasons for rehospitalization were urinary tract infections (UTI), respiratory infections, pressure injuries (PI), and digestive problems [3], all of which are considered preventable [4]. Previous studies have found that race/ethnicity [5, 6], injury severity [2, 6], education [6], employment [5], income [6], and aging [6] are all linked with rehospitalization. Other population-based studies focusing on hospitalization patterns among adult individuals with SCI found that prior-year hospitalizations, chronic health conditions, ambulatory capability, and quality of life indicators [3, 7] also have predictive value.

There is a significant long-term financial burden associated with rehospitalization events, with average and standard deviation total charges of $214,716 and $338,837 per person, respectively, during the first 5 years following injury adjusted to 2019 US dollars [8]. The cost was $65,759 in the first year after injury and ranged from $19,952 to $23,106 per person per year in the subsequent four years [8]. The most significant increase in financial burden was experienced by those with the cervical level of injury, American Spinal Injury Association Impairment Scale (AISA) grades A and B, concomitant injuries, and in-hospital complications [9].

This high prevalence of rehospitalization as well as its associated high cost underscores the need for accurate prediction models to identify individuals at risk, and provide targeted interventions [10]. In addition to prediction, understanding the factors influencing prolonged length of stay (LOS) during rehospitalization is essential for resource allocation and patient management [11].

In this context, machine learning (ML) can provide a comprehensive evaluation of these predictors. It has emerged as a widely adopted approach for developing prediction models due to its ability to effectively capture complex and nonlinear relationships between potential predictors and observational outcomes [12]. Studies [13, 14] have utilized ML techniques to predict length of stay in intensive care units for critically ill patients for various specific diseases, demonstrating promising outcomes. In the case of those with TSCI, ML has been applied to predict prolonged LOS during rehospitalization after injury [15] as well as the prognosis of TSCI including mortality [16]. While existing studies reviewed above have explored risk factors and causes of rehospitalization, our research employs distinct analytical methods, leveraging machine learning techniques. Notably, we emphasized data obtained during the initial rehabilitation care and focused on the first-year post injury, given its heightened likelihood of rehospitalization. Furthermore, our study stands out for its large cohort, featuring a substantial sample size, and relies on more recent data, thereby contributing to the current understanding of rehospitalization dynamics. Consequently, the primary aim of the present study was to develop ML models capable of predicting rehospitalization 1-year post-injury and to identify top predictors of rehospitalization using machine learning techniques. The secondary aim was to identify the predictors of prolonged LOS among those who experienced rehospitalization. The insights gained from this study can guide healthcare providers in identifying high-risk patients, implementing proactive interventions, enhancing patient care, and reducing the burden of rehospitalization as well as prolonged LOS in the TSCI population.

## Methods

### Data source and participants

Data were retrieved from the National Spinal Cord Injury Model Systems Database (NSCID), which is believed to capture data from 6% to 13% of new TSCI cases every year in the United States [1]. Since its inception in 1975, 31 federally funded SCI Model Systems centers have contributed data to the database, including demographics, injury and medical characteristics, and functional independence during the initial rehabilitation and at 1, 5, and every 5 years post-injury [17]. Additional details about NSCID data collection and instructions can be found at: https://www.nscisc.uab.edu/Public_Pages/Database.

Data included patients who were enrolled in the NSCID with a date of injury from 2011 to 2020. To predict rehospitalization 1-year post-injury, the data were divided into 2 groups, rehospitalization and non-rehospitalization, based on the presence of rehospitalization events during year 1 post-injury regardless of their number.

To identify predictors of prolonged LOS, the rehospitalization group was further divided into 2 groups based on total LOS for all rehospitalization events during the first year. Prolonged stay was defined as total LOS greater or equal to the 75th quartile [15].

### Study design and variables

The study variables obtained for initial rehabilitation care included sociodemographic characteristics (age, sex, race/ethnicity, marital status, education, employment, family income, type of insurance, and place of residence), medical history (diabetes, hypertension, arthritis, hyperlipidemia, depression, anxiety disorders, and alcohol use), body mass index (BMI), days from injury to hospital admission, trauma etiology, presence of associated injuries, vertebral injury, spinal surgery, use of mechanical ventilation, method of bladder management, and functional status as assessed by the Functional Independence Measure (FIM). Neurological data were obtained within 7 days of discharge under the International Standards for Neurological Classification of SCI, including motor and sensory scores, level of injury, and AIS.

Information collected at the first anniversary year was used to identify rehospitalization events (yes, no), number of times and days rehospitalized, and reasons for rehospitalization (UTI, PI, and others).

### Statistical analysis

All analyses were conducted using R software. Descriptive statistics for categorical variables are presented as frequencies and percentages, while continuous variables are reported as means with standard deviations (SD). The normality of age distribution was assessed using Shapiro-Wilk test. Group comparisons for continuous variables were performed using Mann-Whitney U test, while Chi-Square test or Monte Carlo method was applied for categorical variables, as appropriate. All tests were two-sided, with a significance level set at 0.05.

### Data preprocessing

Multicollinearity increased variability in the predicted variables, in turn causing inaccurate results for the prediction associated with interrelated explanatory variables [18]. Therefore, we checked it in our independent variables by creating a correlation matrix between them, and for those with coefficients more than or equal to 0.5, one of them was excluded. As a result of that, a high correlation was found between the level of injury (cervical, thoracic, lumbar, sacral) and the severity of disability (Tetraplegia, paraplegia, or normal). Additionally, a high correlation was found between AIS with ASIA motor and sensory total scores. This resulted in dropping the severity of disability and AIS variables.

K-Nearest Neighbor imputer technique [19] was used for replacing missing data for continuous variables. For categorical variables, Frequency Category (mode) Imputation technique was used for imputing missingness, and then one-hot encoding was done. The total LOS were not imputed to avoid bias in model evaluation metrics.

To handle imbalance in sample size between rehospitalized and non-rehospitalized groups as well as between prolonged versus non-prolonged LOS groups in the training dataset, multiple methods were tried and the method that resulted in the best performance for the rehospitalization model was synthetic Minority Oversampling Technique (SMOTE) [20] while for the prolonged LOS model, random oversampling technique [21] was best. The data were split into 80% for the training model and 20% for testing.

### Classification models

After preprocessing, seven frequently utilized machine learning classification algorithms were employed on the dataset. These algorithms encompassed Decision Tree (DT), Support Vector Machine (SVM), Naïve Bayes (NB), Logistic Regression (LR), Random Forest (RF), Adaptive Boosting (Adaboost), and Extreme Gradient Boosting (XGBoost). Optimization of each model’s hyperparameters was conducted through the Grid search technique.

Preprocessing and subsequent analysis were conducted utilizing Python 3.10.5 [22].

### Feature selection and dimensionality reduction

The best-performing model was selected based on resulting accuracy and area under the curve (AUC), and then dimensionality reduction was performed using recursive feature elimination (RFE) [23], and three regularization techniques [24]: Least Absolute Shrinkage and Selection Operation regression, Ridge and Elastic Net regression techniques and the highest AUC resulted from RFE for both rehospitalization and prolonged LOS models. This backward selection method starts with all predictors, iteratively removes the least significant ones, and recalculates importance scores. The final predictor set is chosen based on the subset size that optimizes performance criteria and importance rankings. Finally, this optimal subset of variables is utilized to train the final model [23].

### Evaluation criteria

The evaluation metrics for each model resulted from applying the trained model on the test dataset. We based our assessment primarily on the accuracy score obtained through 5-fold cross-validation to mitigate overfitting concerns. Subsequently, we computed the mean and standard deviation of the results from the five accuracy scores, AUC, F1 score, sensitivity, and specificity. These metrics were determined using the following formulas where TP is true positive, TN is true negative, FP is false positive, and FN is false negative:

Overall accuracy = (TP + TN)/ (TP + FP + TN + FN)

Sensitivity = TP / (TP + FN)

Specificity = TN / (TN + FP)

F1 Score = TP / (TP + ½ (FP + FN))

For the prolonged LOS model, despite addressing the notable imbalance between prolonged and non-prolonged LOS groups through random oversampling, achieving high accuracy alone does not ensure favorable scores in other evaluation metrics like specificity, sensitivity, and F1-score. Consequently, our primary reliance for selecting the optimal model lies on the Area Under the Curve (AUC) scores, complemented by accuracy scores [25].

### Model interpretation

SHapley Additive explanations (SHAP) is the average marginal contribution of a variable considering all possible combinations [26]. They were calculated to identify the highest predictors of rehospitalization and their effect on model outcomes.

## Results

Among the total 4961 study participants, 1704 (34.3%) experienced one or more rehospitalization events during the first post-injury year. Of those 1704 rehospitalized, 421 (24.7%) participants had a prolonged stay (≥17 days), 1254 (73.6%) participants had a non-prolonged stay, and the rest 29 (1.7%) were excluded from analysis because of unknown LOS.

Table 1 displays baseline characteristics of the participants who were followed up for one-year post-injury and they were categorized into rehospitalized versus non-rehospitalized. The mean age was about 42 years, 79% were males and around 30% were minority race. The majority was discharged to a private residence. Around two-thirds of each group was working and had a high school degree or less.

**Table 1:** Demographic characteristics of study participants.

Characteristics	Non- rehospitalized	Rehospitalized	Test of significance^a^
	(*n* = 3257)	(*n* = 1704)	(*p*-value)
Age at injury			
Mean (SD)	41.5 (18.0)	42.8 (17.4)	0.003^b^
Median [IQR]	39.0 [25, 56]	42.0 [27, 57]	
Sex			
Male	2573 (79.0)	1353 (79.4)	0.914
Female	681 (20.9)	350 (20.5)	
Other, Transgender	3 (0.1)	1 (0.1)	
Place of residence at discharge			
Private	3017 (92.6)	1484 (87.1)	<0.001^b^
Nursing Home	158 (4.9)	159 (9.3)	
Hospital	43 (1.3)	30 (1.8)	
Assisted Living	12 (0.4)	13 (0.8)	
Other	12 (0.4)	6 (0.4)	
Race			
White	2264 (69.5)	1180 (69.2)	0.152
Black	726 (22.3)	407 (23.9)	
Other	240 (7.4)	100 (5.9)	
Marital status at injury			
Single	1493 (45.8)	706 (41.4)	0.001^b^
Married	1229 (37.7)	652 (38.3)	
Other	528 (16.2)	338 (19.8)	
Type of insurance at injury			
Private	1733 (53.2)	824 (48.4)	0.004 ^b^
Medicare/Medicaid	1087 (33.4)	649 (38.1)	
Other	382 (11.7)	196 (11.5)	
Occupation at injury			
Working	2127 (65.3)	1082 (63.5)	<0.001^b^
Not working	768 (23.6)	471 (27.6)	
Student	305 (9.4)	105 (6.2)	
Other	47 (1.4)	38 (2.2)	
Level of education at injury			
High school or less	2111 (64.8)	1150 (67.5)	0.120
Associate or Bachelor	799 (24.5)	366 (21.5)	
Postgraduate studies	277 (8.5)	149 (8.7)	
Other/unknown	70 (2.1)	39 (2.3)	
Reasons of rehospitalization^c^			
Urinary tract infection	-	630 (36.9)	-
Pressure injuries	-	192 (11.3)	
Other causes	-	882 (51.8)	
Total LOS (*n* = 1675)^d^			
Mean (SD)	-	17.5 (36.2)	-
Median [IQR]	-	7 [3, 17]	

^a^Test of significance for age is Mann Whitney test, for sex is Monte Carlo test and for the rest of variables is Chi-Square. All tests are 2-sided.

^b^Statistically significant (p-value ≤ 0.05).

^c^Reasons of rehospitalization were created by checking reason of hospital admission at each rehospitalization event in the first-year post-injury and merging them on one variable.

^d^The TLOS information is unknown for 29 participants and LOS was not imputed for these participants because it is the outcome to predict using a specific set of variables in the model. If imputing the missing values and then using those imputed values within the same dataset to test the prediction, it could introduce bias.

The average rehospitalization events was 1.7 ± 1.1 times ranging from 1 to ≥7 times during the first year after injury. One-third of rehospitalized participants had at least one readmission during the first year post-injury because of UTI (36.9%) followed by PI (11.3%) (Table 1).

Table 2 presents the performance of seven machine learning classification models for predicting rehospitalization. Among these models, SVM, DT, and NB had the lowest accuracy rates, followed by LR. The ensemble models—RF, Adaboost, and XGBoost—performed better in terms of accuracy. In terms of sensitivity, SVM had the lowest ability to correctly predict rehospitalized patients (61.4%), while NB had the highest (79.9%). For all seven models, specificity exceeded sensitivity, with SVM showing the lowest specificity and RF the highest.

**Table 2:** Performance of machine learning models in the prediction of rehospitalization during the first-year post-TSCI.

Classification model		Accuracy score (5-fold cross-validation)	F1 score	Sensitivity (Recall)	Specificity	Area under curve (AUC)
Support Vector Machine		63.4 ± 0.6%	62.5%	61.4%	63.7%	62.6%
Decision Tree		63.0 ± 2.1%	66.4%	67.0%	64.2%	65.6%
Naïve Bayes		59.2 ± 2.4%	70.7%	79.9%	52.6%	66.3%
Logistic regression		69.8 ± 2.7%	67.6%	63.2%	75.2%	69.3%
Extreme gradient boost (XG Boost)		72.7 ± 1.0%	73.2%	69.4%	80.1%	74.4%
AdaBoost		74.2 ± 2.0%	73.6%	69.4%	78.7%	74.8%
Random forest^a^	Full model (53)	72.0 ± 2.1%	75.8%	72.5%	80.7%	76.6%
	Reduced model^b^ (26)	75.5 ± 1.1%	75.7%	73.4%	79.0%	76.2%

^a^The best model to be used is the Random Forest classification model.

^b^Reduced after doing dimensionality reduction using Recursive Feature Elimination (RFE).

Most evaluation metrics were highest in the RF model (best-performing model). After performing RFE for RF model, the accuracy and sensitivity increased, while all other evaluation metrics remained almost the same or slightly decreased.

In the prolonged LOS model, Table 3 indicates that DT and NB had the lowest AUC, followed by SVM, LR, and XGBoost, with Adaboost achieving the highest AUC. Applying RFE to the last model improved all evaluation metrics.

**Table 3:** Performance of machine learning models in the prediction of prolonged LOS during rehospitalization the first year post TSCI.

Classification model		Accuracy score (5-fold cross-validation)	Sensitivity (Recall)	Specificity	Area under curve (AUC)
Decision Tree		61.8 ± 5.9%	30.3%	78.4%	55.1%
Support Vector Machine		77.3 ± 0.6%	43.4%	64.1%	58.4%
Naïve Bayes		30.1 ± 3.2%	14.5%	91.9%	55.1%
Logistic regression		74.3 ± 4.5%	53.9%	68.3%	62.1%
Random forest		77.3 ± 0.6%	21.1%	93.8%	59.5%
Extreme gradient boost (XG Boost)		70.1 ± 2.8%	34.2%	88.4%	58.0%
AdaBoost^a^	Full model (55)	69.6 ± 2.4%	55.3%	68.3%	61.8%
	Reduced model ^b^ (27)	66.9 ± 2.0%	59.2%	70.3%	64.7%

^a^The best model to be used is the Ada-boost classification model.

^b^Reduced after doing dimensionality reduction using Recursive Feature Elimination (RFE).

**Fig. 1:**
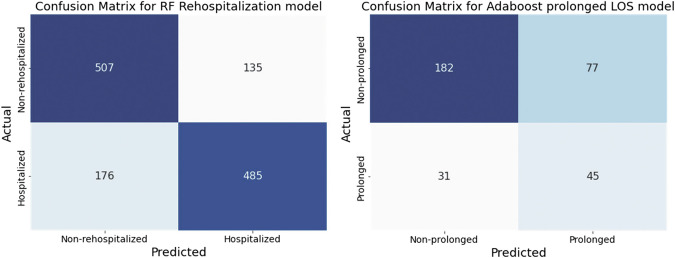
Confusion matrix for performance evaluation of Rehospitalization and prolonged LOS prediction models.

Figure 1 shows the confusion matrices for the best-performing models in rehospitalization and prolonged LOS prediction after RFE.

The selected variables for building the rehospitalization RF and prolonged LOS model based on RFE dimensionality reduction were summarized in Table 4.

**Table 4:** The rank-one variables in Random Forest Rehospitalization and Adaboost prolonged LOS models after Recursive feature elimination.

Variables			RF Rehospitalization model	Adaboost prolonged LOS model
*Socio-demographic variables at time of injury*				
Marital status	Married		√	-
	Single		√	-
Residence	Private		-	√
	Hospital		-	√
	Assisted living		-	√
Gender			√	√
Family income			√	√
Type of insurance	Medicare/Medicaid		√	-
	Private		√	-
Age at time of injury			√	√
Occupation	Working		√	-
Education	High school or less		√	-
	Associate or Bachelor		√	-
*Clinical and neurological assessment*				
Body Mass index (BMI)			√	√
Number of Days from Injury to initial rehabilitation admission.			√	√
Etiology of trauma	Vehicular		√	√
	fall or hit by flying object		√	-
	Violence		-	√
	Sport/Recreation		-	√
Performance of spinal surgery during initial hospitalization			√	√
Associated vertebral injury			√	√
Associated injuries with SCI event			√	√
Level of spinal injury		Lumbar	-	√
		Sacral	-	√
Anal reflexes		Voluntary anal contraction	√	√
		Deep anal pressure	√	-
ASIA Motor score			√	√
ASIA sensory scores		Pin Prick	√	√
		Light Touch	√	√
Bladder management using intermittent catheterization program.			√	-
Functional Independence Measure (FIM) total motor score.			√	√
*Medical history one-year prior to injury*				
Diagnosis of depression by a health professional prior to the SCI.			√	√
Anxiety diagnosed by a health professional.		Panic disorder	-	√
		PTSD	-	√
		Multiple diagnosis	-	√
the number of times a participant drank alcohol during the year before spinal cord injury			√	-
*Reasons of rehospitalization*				
Pressure Injuries (PI)			N/A	√
Urinary tract Infection (UTI)			N/A	√

**Fig. 2:**
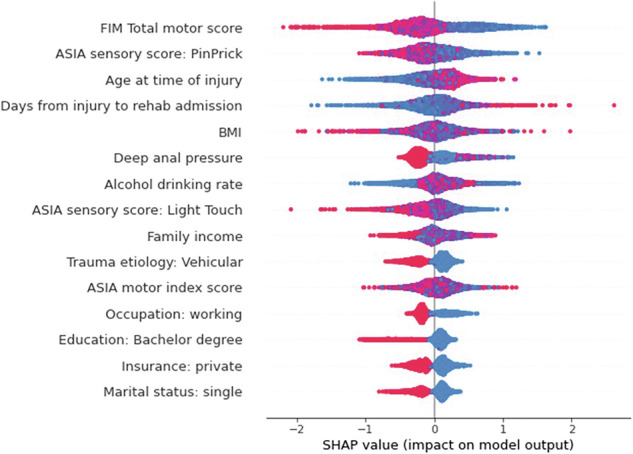
SHAP summary (bee swarm) plot showing variables’ importance for rehospitalization prediction. ^a^FIM Functional Independence Measure, ASIA American Spinal Injury Association, BMI Body Mass Index.

**Fig. 3:**
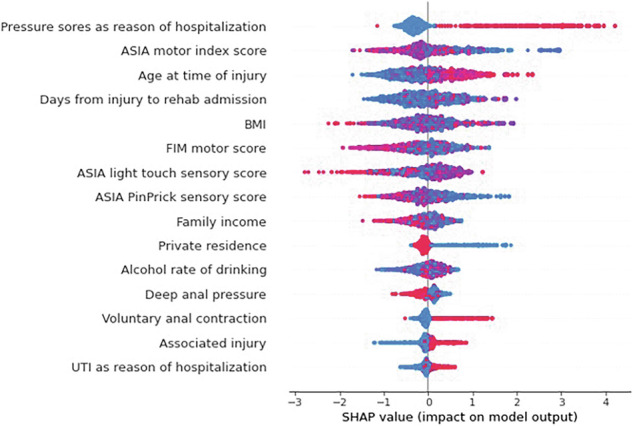
SHAP summary (bee swarm) plot showing variables’ importance for prolonged LOS prediction. ^a^ASIA American Spinal Injury Association, BMI Body Mass Index, FIM Functional Independence Measure, UTI Urinary Tract Infection.

The SHAP values of the most important variables in model prediction were plotted (Figs. 2, 3). The summary plot arranges the variables based on their SHAP value magnitudes in a descending manner. These values are then utilized to illustrate how the first 15 variables impacted the model output. Color-coded representation is employed to signify the feature values, where red indicates high values and blue indicates low values. A SHAP value positioned to the right of the midline denotes a positive impact on prediction, while a value to the left indicates a negative impact. The plot displays the variables in order of their predictive influence and demonstrates how varying values of each variable influenced the prediction outcome [26].

Figure 2 shows the top 15 variables that have the highest SHAP values for rehospitalization prediction. For the sociodemographic characteristics, the higher the family income, the older age at time of injury, being working, bachelor’s degree and private insurance, and being single were associated with lower risk of rehospitalization. Regarding clinical and neurological assessment, the higher the FIM total motor score, ASIA motor and sensory scores, and preservation of deep anal pressure were also associated with reduced risk.

In the prolonged LOS model, it is shown in Fig. 3, that presence of PI as a reason for rehospitalization is a top predictor. Similar to rehospitalization predictors, FIM and ASIA scores, age at time of injury, BMI, rate of alcohol drinking one-year prior injury and time lapse between time of injury to initial rehabilitation admission were considered important predictors.

## Discussion

The primary aim of the present study was to use machine learning techniques in the prediction of rehospitalization during the first year after TSCI based on personal sociodemographic and health data at the time of injury, and clinical characteristics at the time of initial discharge. The seven most used ML classification models were tried based on using all variables and their performance metrices reported. The best-performing model was RF. This algorithm consists of many individual decision trees that operate as an ensemble. Each individual tree in the random forest produces a class prediction, and the class with the most votes becomes the model’s prediction This technique may explain its superior performance compared to the other applied six models.

The RF model was trained and validated resulting in a predictive model with the ability to truly forecast 75.5 ± 1.1% of all predications based on 26 variables that were all non-invasive in nature and were routinely collected on the patients’ initial hospitalization, therefore, can efficiently be deployed in clinical settings. This final model can effectively identify a significant proportion (73.4%) of patients who are likely to experience rehospitalization (sensitivity), enabling timely intervention and targeted support for these individuals. It also has an AUC of 76.2%, which means a moderate to strong ability to distinguish between patients who will require rehospitalization versus those who will not. This discriminative power could help clinicians set individualized follow-up policies for each patient.

A study conducted by Howard et al. [27]. in 2021 used ML to predict rehospitalization only within first 90days after injury but it is categorized by 3 post-acute care discharge sites: home with home care services (HC), skilled nursing facility (SNF), and inpatient rehabilitation facility (IRF). The study found that patients generally experience lower rehospitalization rates, when discharged to SNF, with the exception being those classified in the very high-risk group. Another study examined factors associated with early rehospitalization among patients discharged to post-acute facilities [28]. It found that patients rehospitalized within the first week tended to be of older age, white, residing in urban areas, exhibiting fewer concurrent medical conditions, having a history of multiple prior hospital admissions, and less frequently covered by Medicare insurance [28]. This is consistent with our finding regarding the older age and type of insurance as important predictors based on SHAP.

Our RF model’s SHAP results indicated that a higher FIM motor score at discharge was associated with a lower predicted risk of rehospitalization. This supports the results from a multivariate logistic regression in a multicenter study reporting that lower motor FIM was related to rehospitalization (odds ratio [OR] 0.978, P < 0.001). Their results were similar to our finding and it suggested that people discharged with lower motor FIM scores will have a higher likelihood of rehospitalization [10]. Similarly, another study reported that the FIM motor score is lower among re-admitted TSCI patients compared to not readmitted [29].

Our study showed that older age at the time of injury will increase the SHAP value for the prediction and this could be attributed to factors such as increased comorbidities with age, slower recovery rates, and greater vulnerability to complications following SCI. Cardenas et al. [10] documented that there were no significant differences in the rate of rehospitalization among different age groups. This difference may be attributed to smaller sample size especially in the younger age group for this comparative study.

Moreover, another study focused on healthcare utilization after TSCI found that factors predicting multiple hospital stays were urban living, age 60 years or older, and low FIM score at time of discharge while factors predicting emergency department (ED) rehospitalization were rurality, low income as well as low FIM score [30]. Another study reported that income, education, and age had significant bivariate relationships with rehospitalization [6]. These findings were consistent with the top predictors that we got in SHAP plot (Fig. 2).

For prediction of prolonged LOS, our Adaboost model shows moderate performance, with an accuracy of 66.9% and a sensitivity of 59.2%, suggesting it misses some patients likely to experience prolonged stays. However, its specificity of 70.3% indicates a reasonable ability to avoid overestimating the need for prolonged LOS, and an AUC of 64.7% suggests moderate discriminative ability. Similarly, Fan et al. [15]. built an ensemble classifier model with AUC of 79.9% to predict prolonged LOS which was >14 days based on the distribution of LOS in their data. However, they used different variables for building their model.

By examining predictors, we found that the top predictor was PI as a reason for prolonged LOS. Moreover, similar to our study, Wu et al. [11] found that prolonged length of stay was significantly associated with age, trauma etiology, ASIA impairment scale, associated injuries, and spinal surgery. However, the current observed assessments are insufficient to provide an acceptable level of predictive performance. Hence, the NSCISC may need to explore additional assessments to help in prolonged stay risk prediction.

### Limitations

Generally, The NSCID has limitations that must be considered when evaluating the study results. The generalizability of the present study findings is limited, given a hospital-based sample, selective eligibility, change in the designation of SCIMS centers over time, and exclusion of non-traumatic SCI and those who did not receive inpatient care after TSCI.

For the prolonged LOS model, there was a major difference between the number of participants in 2 classes, affecting the model evaluation metrics. Moreover, the variables collected at the time of discharge performed less well when used for predicting prolonged LOS compared to rehospitalization, and the highest AUC that we calculated was only 64.7% which was relatively low compared to previously published models, so more variables need to be tested to be able to deploy in clinical settings.

For the rehospitalization model, while the evaluation metrics are relatively strong, the use of 26 variables in the prediction process may introduce complexity from a clinical application standpoint.

## Conclusion

The resulting RF model from this study had moderate discriminative accuracy (76.2%) in distinguishing between rehospitalized and non-rehospitalized patients based on sociodemographic and clinical characteristics at initial discharge from the rehabilitation center after TSCI. An added value is that the variables used are noninvasive data that are collected routinely, earliest in the patient’s initial admission process. We found that the most important clinical variables are higher FIM motor scores, ASIA sensory and motor scores, the presence of deep anal pressure, higher educational degrees, occupation, having private insurance, alcohol drinking rate, higher BMI, the time gap from injury to admission, and family income for predicting a decreased risk of rehospitalization. Among rehospitalized patients, factors such as PI, UTI, low FIM and ASIA scores, older age at the time of injury, and associated injuries with SCI at the time of initial rehabilitation were identified as contributors to prolonged LOS, though the predictive performance of the model was limited.

### Clinical significance

The rehospitalization model provides valuable insight at the time of discharge, allowing clinicians to identify high-risk patients and implement timely, personalized interventions. By helping in preventing unnecessary rehospitalizations, the model may support improved patient outcomes and more efficient healthcare delivery. The prolonged LOS model, despite its limitations, can help inform care plans and resource allocation, enabling a more holistic approach to TSCI patient management.

## Supplementary information

Dataset source

## Data Availability

The deidentified data is publicly available through NSCISC website: https://www.nscisc.uab.edu/Research/NSCISC_DatabasePublicUse.

## Code availability

Code was written in Python programming language using scikit-learn libraries and is available upon request.
