# Supplementary material for: Utilization of machine learning algorithm in the prediction of rehospitalization during one-year post traumatic spinal cord injury
Source: Spinal Cord. 2025 Feb 15;63(4):214–21. doi: 10.1038/s41393-024-01055-9 (PMC12003193; doi:10.1038/s41393-024-01055-9)
Supplement: Supplementary file 1 — Dataset source [file 41393_2024_1055_MOESM1_ESM.pdf]

The Access for dataset used in the analysis

- The deidentified data is publicly available through NSCISC website:

https://www.nscisc.uab.edu/Research/NSCISC_DatabasePublicUse
